# Epigenetically regulated *PCDHB15* impairs aggressiveness of metastatic melanoma cells

**DOI:** 10.1186/s13148-022-01364-x

**Published:** 2022-11-28

**Authors:** Arnaud Carrier, Cécile Desjobert, Valérie Lobjois, Lise Rigal, Florence Busato, Jörg Tost, Miquel Ensenyat-Mendez, Diego M. Marzese, Anne Pradines, Gilles Favre, Laurence Lamant, Luisa Lanfrancone, Chantal Etievant, Paola B. Arimondo, Joëlle Riond

**Affiliations:** 1 Unité de Service et de Recherche USR n°3388 CNRS-Pierre Fabre, Epigenetic Targeting of Cancer (ETaC), Toulouse, France; 2 Cancer Epigenetics Group, Institut de Recerca Contra la Leucèmia Josep Carreras, Barcelona, Spain; 3 Institut des Technologies Avancées en Sciences du Vivant – ITAV-USR3505, CNRS, Université de Toulouse, Université Paul Sabatier-UT3, Toulouse, France; 4 Laboratoire de Biologie Cellulaire et Moléculaire du Contrôle de la Prolifération, CNRS UMR 5088, Université Paul Sabatier-UT3, Toulouse, France; 5 Laboratory for Epigenetics and Environment, Centre National de Recherche en Génomique Humain, CEA-Institut de Biologie Francois Jacob, Université Paris-Saclay, Evry, France; 6 Cancer Epigenetics Laboratory at the Cancer Cell Biology Group, Institut d’Investigació Sanitària Illes Balears (IdISBa), Palma, Spain; 7 Inserm, CNRS, Centre de Recherches en Cancérologie de Toulouse, Université de Toulouse, Université Toulouse III-Paul Sabatier, Toulouse, France; 8 Laboratoire de Biologie Médicale Oncologique, Institut Claudius Regaud, Institut Universitaire du Cancer de Toulouse-Oncopole, Toulouse, France; 9 Laboratoire d’Anatomopathologie, Institut Universitaire du Cancer Toulouse Oncopole, Toulouse, France; 10 Department of Experimental Oncology, Instituto Europeo di Oncologia, Via Adamello 16, 20139 Milan, Italy; 11 EpiCBio, Epigenetic Chemical Biology, Department Structural Biology and Chemistry, CNRS UMR N°3523, Institut Pasteur, 28 Rue du Dr Roux, 75015 Paris, France

**Keywords:** DNA methylation, Aggressiveness, Melanoma, Tumor suppressor, Protocadherin

## Abstract

The protocadherin proteins are cell adhesion molecules at the crossroad of signaling pathways playing a major role in neuronal development. It is now understood that their role as signaling hubs is not only important for the normal physiology of cells but also for the regulation of hallmarks of cancerogenesis. Importantly, protocadherins form a cluster of genes that are regulated by DNA methylation. We have identified for the first time that *PCDHB15* gene is DNA-hypermethylated on its unique exon in the metastatic melanoma-derived cell lines and patients’ metastases compared to primary tumors. This DNA hypermethylation silences the gene, and treatment with the DNA demethylating agent 5-aza-2′-deoxycytidine reinduces its expression. We explored the role of *PCDHB15* in melanoma aggressiveness and showed that overexpression impairs invasiveness and aggregation of metastatic melanoma cells in vitro and formation of lung metastasis in vivo. These findings highlight important modifications of the methylation of the *PCDHβ* genes in melanoma and support a functional role of *PCDHB15* silencing in melanoma aggressiveness.

## Introduction

Melanoma is a type of cancer with increasing incidence [1] and, until recently, was often fatal once it metastasized to distant organs. New therapeutic approaches include the molecular targeting of activated oncogenes and immune-based therapies, even in patients with advanced disease [2]. Nevertheless, many patients develop therapy resistance or do not respond to treatment. Therefore, the identification of molecular traits underpinning melanoma aggressiveness remains an ongoing challenge not only to improve treatment, but also to improve diagnosis and prognosis [3].

Besides the activating mutations in the *BRAF* and *NRAS* oncogenes, found in significant proportions of primary melanomas, important epigenetic changes occur in melanoma. These modifications include in particular aberrant DNA methylation of cytosine (5-methylcytosine (5mC)) at CpG sites—including both hyper- and hypomethylation, loss of 5-hydroxymethylcytosine (5hmC), histone modifications and ncRNA expression [4–6]. Several studies have associated DNA methylation changes with melanoma initiation and progression [7–10] and genome-wide analysis correlated DNA methylation signatures and silenced genes to different melanoma stages [11–19]. We have previously provided evidence supporting that aberrant DNA methylation regulates genes involved in melanoma progression and aggressiveness by identifying a microRNA, miR-199a-3p, regulated by DNA methylation and whose up-regulation led to reduced tumor cell invasion in vitro and in vivo [20]. Next, we used a multi-step strategy to identify the aberrant DNA methylation patterns that characterize human melanoma aggressiveness independently of the physiological background [21]. Among the aberrant methylated CpGs patterns that mark melanoma aggressiveness in patient primary tumors, we found the *PCDHB15* gene. This gene belongs to a cluster encoding for adhesion molecules, the protocadherins, related to the cadherin superfamily. Some protocadherins are predominantly expressed within the central nervous system during development, suggesting important neurobiological roles. Others, expressed in tissues at adult stages, seem to regulate cellular differentiation, tissue regeneration and maintenance. Interestingly, while their functional role remains mostly elusive, loss of protocadherins has been linked to several cancer types [22]. In particular, a region of 800 kb, which includes protocadherins α and *γ* families, was reported to display long-range epigenetic silencing (LRES) in breast cancer [23], Wilm’s tumor [24] and colorectal cancer [25]. In neuroblastoma, aberrant DNA methylation of the *PCDHB* family was proposed as part of the CpG island methylator phenotype (CIMP) [26] and was strongly associated with poor prognosis [27–29].

Here, we show that *PCDHB15,* a member of this cluster of genes, marks melanoma aggressiveness and plays a functional role in regulating the hallmarks of cancerogenesis. We observed that *PCDHB15* is hypermethylated at the 5′ end of its unique exon and is not expressed in two metastatic melanoma-derived cell lines, WM266-4 and WM983A. TCGA data confirm that *PCDHB15* hypermethylation is observed in patient metastasis samples compared to primary tumor samples. Interestingly, the expression of this gene was modulated upon treatment with the DNA demethylating agent 5-aza-deoxycytidine (5azadC). In addition, overexpression of *PCDHB15* impaired metastatic melanoma cell invasiveness and aggregation in vitro*,* and metastasis formation in vivo. For the first time, our findings support a potential role of *PCDHB15* silencing contributing to melanoma aggressiveness by important DNA methylation modifications of the gene.

## Material and methods

### Cell culture

The WM115 and WM266-4 cells, as well as WM983A and WM983B cells, were established from a primary VGP melanoma and metastasis from the same patient, respectively [30]. In vitro, the cell lines with metastatic origin (WM266-4, WM983B) displayed a higher invasive potency, compared to cells from primary melanomas (WM115, WM983A), as assessed in 3D spheroids invasion assays [31] and human reconstructed skin models [32–34].

The WM266-4 and WM115 cells (obtained from the American Type Culture Collection) were grown in DMEM (Invitrogen, France) supplemented with 10% fetal bovine serum (Sigma, France), 2 mM glutamine, 100 UI/mL penicillin–streptomycin, and in a 5% CO_2_ atmosphere. The WM983A and WM983B cells (purchased from the Coriell Institute) were grown in MCDB153 medium with 20% Leibovitz’s L-15 medium (v/v), 2% FBS heat-inactivated (v/v), 5 μg/mL insulin and 1.68 mM CaCl_2_. The numerations of viable cells were performed using an Automated Cell Viability Analyzer (Beckman Coulter Vi-Cell).

### Establishment of stable cell lines

WM266-4 cells were seeded at 6 × 10^5^ cells in 60 mm dishes and transfected 24 h later using Lipofectamine 2000 (Invitrogen) with 1 µg of the pCMV6-*PCDHB15* plasmid (DDK-tagged *PCDHB15*, RC207719, CliniSciences) or the pCMV6-MOCK plasmid corresponding to the same plasmid without the *PCDHB15* cDNA sequence (obtained from the pCMV6-*PCDHB15* plasmid by digestion with by EcoRI and XhoI, and self-ligation with a linker). The selection of transfected cells was performed in a medium containing 0.8 mg/mL of Geneticin (Gibco). Cell lines expressing *PCDHB15* were established from 3 of 15 isolated clones. PCDHB15 expression was characterized by RT-qPCR. The control cell line (WM266-4 MOCK) is a pool of transfected cells with the pCMV6-MOCK plasmid. Transfected cells were maintained in culture in a medium containing 0.6 mg/mL Geneticin for 10 passages. These modifications did not impact morphology proliferation and viability. All experiments were conducted under 20 cell passages in culture.

### Tumor samples

Tumor samples from four melanoma patients were retrieved from the tumor tissue bank at the Department of Pathology, IUCT-O Toulouse Hospital (France). The study was carried out in accordance with the institutional review board-approved protocols (CRB, AC-2013-1955), and the procedures followed the Helsinki Declaration. Pathological specimens consisted of frozen samples from primary (*n* = 13) and metastasis samples (*n* = 9). Additional frozen primary melanoma samples (*n* = 5) were provided by the Department of Experimental Oncology, European Institute of Oncology, Milan (Italy).

### Cells treatment with 5-aza-2′-deoxycytidine (5azadC)

5-aza-2′-deoxycytidine (5azadC, decitabine) was bought from Sigma-Aldrich (France) and dissolved in acidic water at 10 mM and stored in single-use aliquots at − 20 °C.

WM266-4 cells were seeded at the density of 6 × 10^6^ cells per 75 cm^2^ flasks (day 0) and treated with 5azadC after a 12 h period to allow cell attachment and synchronization in G0/G1 phase. Cells were treated daily for 72 h (day 1, 2, 3) at the indicated concentration of 5azadC. They were collected at day 4 for analysis of DNA methylation patterns by pyrosequencing and day 7 for expression analyses.

### Genomic DNA isolation

Genomic DNA from cell lines was isolated using the DNeasy Tissue kit (Qiagen, France). Genomic DNA from patients’ samples was isolated using the QiaAmp kit (Qiagen, France).

### Bisulfite pyrosequencing

Quantitative DNA methylation analysis was performed by pyrosequencing of bisulfite-treated DNA as previously described [35]. Sequences including CpGs were amplified using 20 ng of bisulfite-treated human genomic DNA and 5–7.5 pmol of forward and reverse primer, one being biotinylated. Two pairs of PCR primers were designed for PCR1 (CpG 1, 2, 3 and 4) and PCR2 (CpG 5 and 6). PCR was designed around the hypermethylated probes from previous Illumination 450 k Bead Chip analysis [21].

PCR1: Biotin-TTTAGAGTTGGTGTTGGATATAGAA (Forward) and CCAAAACCAAAATAAAAATCTAAAC (Reverse);

PCR2: TTTAGATTTTTATTTTGGTTTTGGA (Forward) and Biotin-TATAATATCTCTCCATTTATCCCAATATCT (Reverse).

Reaction conditions were 1 × HotStar® Taq buffer (Qiagen) supplemented with 1.6 mM MgCl_2_, 100 μM dNTPs and 2.0 U HotStar Taq polymerase (Qiagen) in a 25 μL volume. The PCR program consisted of a denaturing step of 15 min at 95 °C, followed by 50 cycles of 30 s at 95 °C, 30 s at 60 °C and 20 s at 72 °C, with a final extension of 5 min at 72 °C. A total of 10 μL of PCR product was rendered single-stranded as previously described and 4 pmol of the respective sequencing primers were used for analysis. Quantitative DNA methylation analysis was carried out on a PSQ 96MD system with the PyroGold SQA Reagent Kit (Qiagen) and results were analyzed using the PyroMark software (V.1.0, Qiagen).

### TCGA DNA methylation data analysis

The TCGA-SKCM DNA methylation data was downloaded from GDAC Firehose Broad [36] on February 2021. Normalized beta values for the Illumina probes nearby the *PCDHB15* gene were selected for comparative analyses. DNA methylation for primary melanoma (PRM), lymph node metastasis (LNM), and distant organ metastasis (DOM) was summarized using the mean value and the standard error of the mean. Differential DNA methylation was assessed by the Wilcoxon Rank-Sum test in *R*. All *p* values from multiple comparisons (> 50 tests) were corrected using the False discovery rate (FDR) method. The R/ggplot2 package was used for data visualization.

### mRNA quantification

RNA was purified using the RNeasy Mini Kit (Qiagen, France) and quantified on a NanoDrop2000 (Thermo Fisher Scientific).

Quantification of *PCDHB15* mRNA was performed by RT-qPCR. Total RNA (2 µg) was reverse transcribed into cDNA with the iScript cDNA Synthesis Kit (Bio-Rad, USA). Real-time PCR was performed according to the manufacturer’s recommendations, using SsoAdvanced™ SYBR® Green Supermix (Bio-Rad). The primers were: AGCAGGCCGAGCTCAGATTA (forward) and ATTGGCGTCCAAGACCAAGA (reverse). A CFX384 Touch™ Real-Time PCR Detection System from Bio-Rad (Marnes-la-Coquette, France) was used to run the following PCR program: 95 °C 10 min followed by 40 cycles of 15 s at 95 °C, 30 s at 65 °C for elongation, ended with a fusion cycle to determine the Tm of each amplification product.

The PCR data were analyzed with the CFX Manager v3.0 software (Bio-Rad) to generate the Ct values. The following quality controls were applied: amplification of a single product, no amplification in the NRT (No reverse transcription) condition, efficiency close to 100%, *R*^2^ > 0.98 and SD between technical triplicates < 0.3. The 2 − ΔΔCt method was used to generate the gene expression ratios by amplification of TBP (TATA box binding protein) TTGACCTAAAGACCATTGCACTTCGT (Forward) and TTACCGCAGCAAACCGCTTG (reverse) as normalizing control and data were presented as mRNA fold change of target RNA.

### Western blot analysis

Total protein extract was obtained from confluent cells grown in 75 cm^2^ flasks. The cells were lysed in protein extraction buffer (10 mM Tris HCl, 120 mM NaCl, 1% NP40, 1 mM EDTA, 1 mM DTT and 1X proteases inhibitor (Complete™, EDTA-free Protease Inhibitor Cocktail, Sigma-Aldrich)). Samples were separated on 10% SDS-PAGE gels and transferred onto polyvinylidene difluoride membranes. After saturation with 5% dry milk in Tris NaCl 1% Tween 20, membranes were incubated with either anti-PCDHB15 antibody (NBP1-87322, Novus Biologicals), anti-DDK antibody (4C5, TA50011, OriGene) (1/1000 diluted in 5% dry milk in Tris NaCl 1% Tween 20) or anti-*β* actin antibody (MAB1501, Millipore, 1/1000 in 5% dry milk). After washes, the membranes were revealed with secondary HRP-coupled antibodies (Sigma-Aldrich). The signals were detected by ECL for *β*-actin (GE Healthcare) and Immobilon Western HRP Substrate (Millipore) for PCDHB15 and DDK. The chemoluminescent signals were acquired with a G:BOX imaging system (Syngene).

### PCDHB15 cell surface expression

The expression of PCDHB15 at the cell surface was analyzed by flow cytometry. Cells were detached with 2 mM EDTA in PBS and incubated for 45 min at 4 °C with 1 µg/mL of anti-PCDHB15 antibody (NBP1-87322, Novus Biologicals) in PBS supplemented with 1% BSA. Cells were washed, counterstained with Alexa-647-conjugated goat anti-rabbit Ig antibodies (Invitrogen) and incubated with 0.5 mg/mL DAPI (Sigma). PCDHB15 expression was monitored on live cells (gated as DAPI-negative cells) on a LSRII flow cytometer using the Diva software (both from BD Biosciences, Le Pont-De-Claix, France).

### 3D cell invasion assay

WM266-4 cells were seeded in 96-well plates coated with agarose 1% (Sigma-Aldrich) in PBS (3000 cells in 100 µL medium per well). After 2 days at 37 °C in a 5% CO_2_ atmosphere, cells from one spheroid with a diameter of approximately 300 µm. For each condition, six spheroids were individually embedded in EMEM media (Lonza) containing 1% of bovin collagen I (BD Biosciences) and 2% SVF. Bright-field images from the initial spheroids were acquired with an Axiovert 200 M device (5X Plan-Neofluar objective, Carl Zeiss, Germany). After 24 h at 37 °C, spheroids were labeled 1 h with 2.5 µM calcein (calcein AM, BD Pharmingen) in PBS and fluorescent 6 z-stack images with 20 µm intervals were acquired. The fluorescent pictures were stacked and the total sizes of the spheroids were measured using the Image J (NIH) software. Invasion areas were obtained by subtracting the initial size of the spheroid. The invasion index represents the invasion area at 24 h normalized to the initial spheroid area. If cytotoxic effects appear, the initial spheroid area decrease and the data are not considered.

### Aggregation assay

Cells were dissociated with 2 mM EDTA in PBS and seeded in a CELLSTAR® Cell-Repellent surface 96-well plate (Greiner Bio-One) (500 cells in 100µL medium per well), then centrifuged at 200* g* for 8 min and left at rest for 45 min before time-lapse experiments. Time-lapse video microscopy images were acquired over 20 h (1 acquisition/15 min), by using an inverted widefield Zeiss Axio Observer Z1 microscope fitted with a 0.3 N.A 10 × objective and a CoolSNAP CDD camera (Roper scientific). At each time point and position, 5-µm spaced z-stacks in bright field were acquired using the Meta-Morph software. At each time point, and for each aggregate, areas of the cell aggregates were quantified using an algorithm developed on MATLAB software [37]. The aggregate areas were normalized to the calculated area at the beginning of time-lapse microscopy.

### In vivo metastasis experiments

The animals were handled and cared for in accordance with the Guide for the Care and Use of Laboratory Animals (National Research Council, 1996) and European Directive EEC/86/609, under the supervision of the authorized investigators. Un-anesthetized 7-week-old female SCID mice (ENVIGO RMS SARL, Gannat, France) were injected into the tail vein with 3 × 10^6^ viable cells in 200-μL PBS (WM266-4 WT, WM266-4-pCMV mock or each stable clone overexpressing the *PCDHB15* gene). Groups were constituted of *n* = 15 animals for injection with mock, clone 8 and clone 12; *n* = 14 for clone 13. Twenty-one days after injection, mice were dissected and the organs (except brain) were visually inspected. Lungs only presented detectable metastases. They were recovered, formalin-fixed and paraffin-embedded. Sections were stained with hematoxylin and eosin (H&E). The number and area of metastasis were measured in whole lung sections by immunostaining with Tyrosinase antibody Mob299–05 (1/500) (Diagnostic BioSystems, Pleasanton, CA-USA). 3DHistech (Panoramic 250) was used to scan sections and measure metastases area. Statistics were performed using the Mann–Whitney test.

## Results

### PCDHB15 is hypermethylated in aggressive melanoma cells and patient samples

**Fig. 1:**
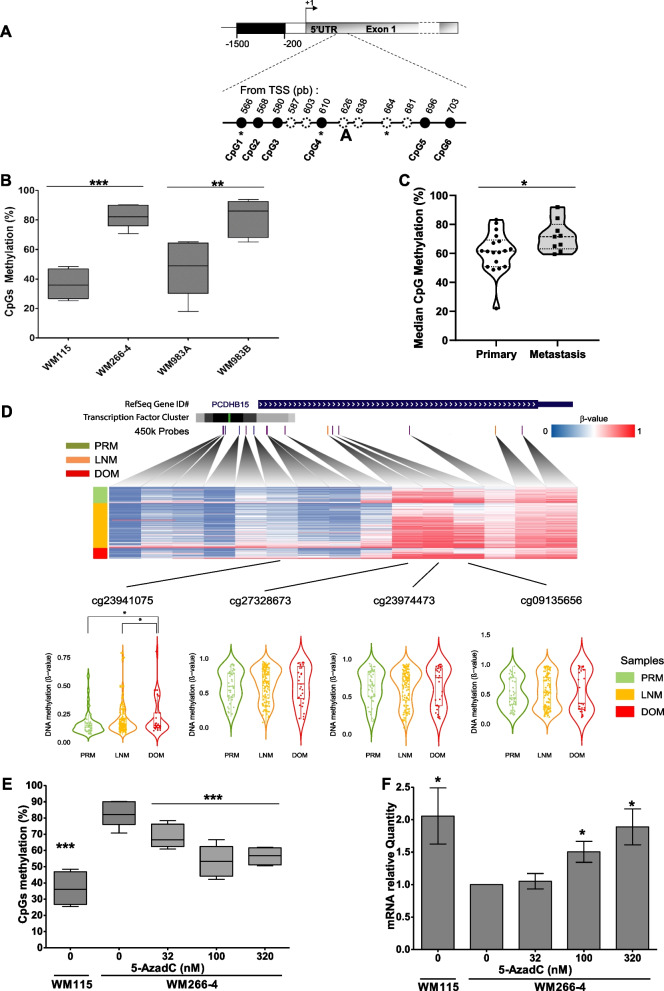
Analysis of CpG methylation of *PCDBH15* in melanoma cell lines and patient samples and re-expression after 5azadC treatment of WM266-4 cell line. **A** The percentage of DNA methylation of each CpG in *PCDHB15* was analyzed by bisulfite conversion followed by pyrosequencing of the CpGs indicated as black dots. CpGs on the sequence but not amplified in pyrosequencing are indicated as dotted lines. The CpGs of the Illumina 450 K array are indicated by an asterisk: for *PCDHB15*, cg27328673, cg23974473 and cg09135656 at + 566, + 610 and + 664pb from the TSS, respectively. **B**–**C** The DNA methylation mean level of *PCDHB15* (**B**) was measured in two pairs of cell lines originating from two different patients, WM115/WM266-4 cells and WM983A/WM983B (**B**), as well as in genomic DNA obtained from 27 patient samples, primary (*n* = 18) or metastases (*n* = 9) (**C**). Data are presented as box plot of the median DNA methylation percentage of CpGs in black (6 CpGs for *PCDHB15).* The median values for primary and metastasis samples are 61.5% and 71.6%, respectively, Jarque–Bera’s test to analyze normality, Fisher’s test to analyze variances and Student *t* test were performed, n.s = not significant, * = *p* < 0.05, ** = *p* < 0.01, *** = *p* < 0.001. **D** Normalized beta values for the Illumina probes of DNA methylation for primary melanoma (PRM), lymph node metastasis (LNM) and distant organ metastasis (DOM) from TCGA-SKCM DNA methylation data of PCDHB15 were summarized as a heatmap. A violin plot was used to highlight CpGs identified as hypermethylated in metastatic cell lines in our previous study. Differential DNA methylation was assessed by the Wilcoxon rank-sum test. All p values from multiple comparisons (> 50 tests) were corrected using the false discovery rate (FDR) method. * = *p* < 0.05. Transcription factor clusters from transcription factor ChIP-seq clusters (340 factors, 129 cell types) from ENCODE 3 were indicated as black/grayscale. WM266-4 cells were treated with increasing concentrations of 5azadC daily during 72 h (d1, d2, d3). **E** At day 4, DNA methylation of *PCDHB15* at exon 1 was measured by pyrosequencing (*n* = 2 for WM266-4 and WM115 cells; *n* = 3 for 5azadC-treated cells). The box plots show the percentage of DNA methylation of the analyzed CpGs (from panel A). **F** The mRNA quantification of *PCDHB15* by RT-qPCR was performed at day 7, using the TBP gene as reference gene and normalized according to the expression level found in the WM266-4 cells (*n* = 4, SEM are shown). Fisher’s test to analyze variances and Student *t* test were performed, ns = not significant, * = *p* < 0.05, ** = *p* < 0.01, *** = *p* < 0.001

By comparing the DNA methylation profile of three highly aggressive metastatic melanoma cell lines (WM266-4, M4BeS2 and TW12) to their less aggressive counterpart derived from the same patient (WM115 and M4Be, respectively) by genome-wide DNA methylation analysis (BeadChip Illumina 450 K, deposited as GSE155856), we identified hypermethylated genes located in gene clusters [21]. Among them, we focused our analysis on *PCDHB15*, which belongs to the protocadherin beta family cluster located on chromosome 5 (5q31.3). In WM115 and WM266-4 cells which are derived, respectively, from the primary tumor and the cutaneous metastasis of the same patient, *PCDHB15* showed differential methylation above 40% in at least two CpGs positions located at + 566 and + 610 pb from the TSS, respectively (Fig. 1A). The differential methylation status in this region was confirmed by pyrosequencing after bisulfite conversion and PCR amplification of six close CpGs, in WM115 and WM266 cells, as well as in the cell line pair WM983A and WM983B derived from the same patient but with different aggressiveness status (Fig. 1B). The boxplots indicate that the DNA methylation median for *PCDHB15* in this region was higher in WM266-4 and WM983B (metastatic) cells compared to WM115 and WM983A (primary) cells, respectively. Interestingly, higher DNA methylation levels were also found in nine patient metastasis samples compared to 18 primary melanoma samples (Fig. 1C). We also investigated the DNA methylation of this gene in The Cancer Genome Atlas database (Fig. 1D), showing a statistically nonsignificant increase in DNA methylation in lymph node (LNM) and distant organ metastasis (DOM) compared to primary melanomas (PRM). Furthermore, a CpG (probe cg24941075) located close to the PCDHB15 promoter region in a transcription factor region (CTCF) was found to be hypermethylated in several DOM patients, leading to a significant difference between DOM *versus* PRM and LNM groups (Fig. 1D).

### DNA hypermethylation of PCDHB15 is associated with decreased gene expression that is reversed upon 5azadC treatment

We then investigated whether the DNA hypermethylation of *PCDHB15* gene 5′-end was associated with gene silencing. The methylation status and expression in WM115 *versus* WM266-4 cells were inversely correlated: WM115 cells, in which *PCDHB15* 5′-end was less methylated than in WM266-4 cells, expressed a twofold higher amount of *PCDHB15* mRNA (Fig. 1E). Treatment of WM266-4 cells with increasing concentrations of the DNA demethylating agent 5azadC for 3 days induced a decrease in DNA methylation of *PCDHB15* in a dose-dependent manner with a plateau at 55% (Fig. 1E). Concomitantly, its expression increased significantly upon treatment with 0.1 µM to 0.32 µM of 5azadC, resulting in a twofold increase compared to the level observed in WM115 cells (Fig. 1F).

These results indicated a potential role for DNA methylation in the silencing of *PCDHB15* correlating with the aggressiveness of metastatic melanoma. We next investigated this hypothesis.

### PCDHB15 overexpression impairs melanoma cells 3D aggregation

**Fig. 2:**
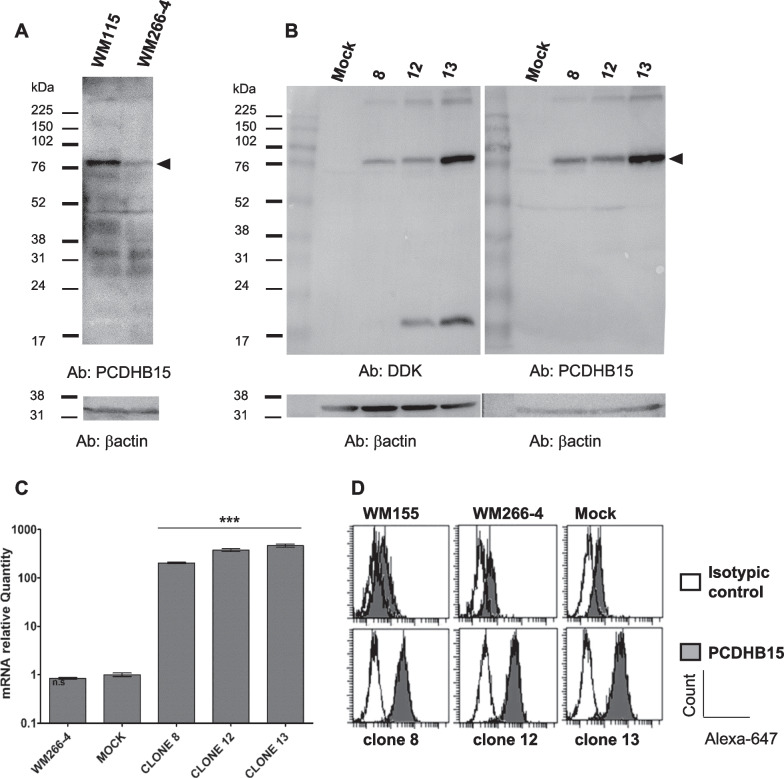
Characterization of PCDHB15-overexpressing clones. **A** Western blot analysis of endogenous PCDHB15 in WM115 and WM266-4 cells and **B** of the overexpression of the PCDHB15 construct in WM266-4 cells, mock (transfected with the empty pCMV6 vector), clone 8, 12 and 13, revealed by the antibody against PCDHB15 or against DDK (for the constructs only). The Western blot is representative of *n* = 3. Beta-actin was used as loading control (bottom). **C** PCDHB15 mRNA quantification by RT-qPCR (on *n* = 3 biologically independent experiments, ANOVA test, *: *p* < 0.05; **:*p*< 0.01. ***: *p* < 0.001). The value in WM266-4 cells was considered as 1. **D** Cell surface expression of PCDHB15 measured by immunolabeling and flow cytometry. Black and white histograms display the cell surface fluorescence associated with PCDHB15 and isotypic control labeling, respectively

*PCDHB15* was overexpressed with a C-terminal DDK-tagged construct in the metastatic WM266-4 cells, in which *PCDHB15* is silenced (Fig. 2A). Three clones overexpressing *PCDHB15* were selected and characterized (clone 8, 12 and 13, Fig. 2B). All three clones produced high levels of *PCDHB15* mRNA compared to mock-transfected and wild-type WM266-4 cells (Fig. 2C), but displayed different content of the full-length protein (Fig. 2B). In addition, a significant amount of protein was detected at the cell surface by cell surface labeling with an anti-PCDHB15 antibody directed against the N-terminal portion of the protein and flow cytometry measurement (Fig. 2D).

**Fig. 3:**
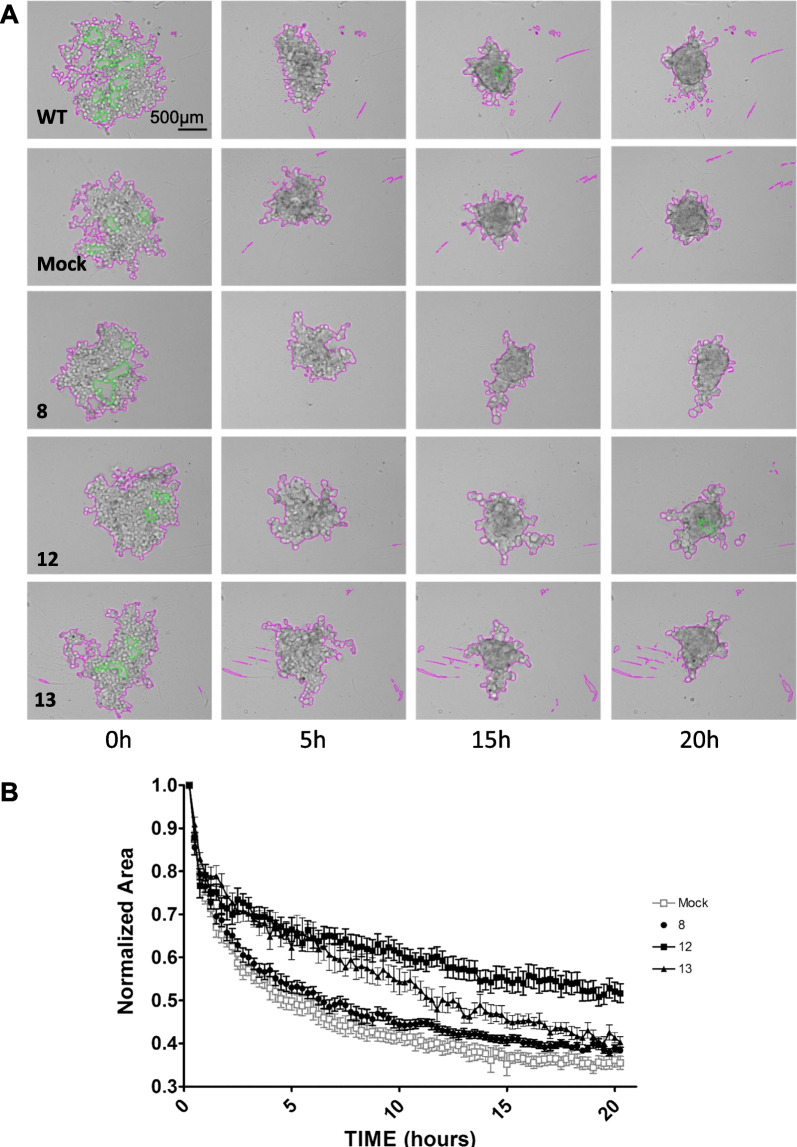
3D aggregation of WM266-4 cells is impaired by PCDHB15 overexpression. The formation of aggregates of WM266-4 cells (WT), control cells (mock) and PCDHB15-overexpressing cells (clones 8, 12 and 13) was monitored by bright-field time-lapse video microscopy. **A** The images show representative aggregates at 0, 5, 15 and 20 h after the experiment onset. Pink lines delineate the maximal aggregate areas. Green lines delineate empty areas that are subtracted in the total area calculation. **B** The normalized area of the aggregates is reported at each time. The reported values are the mean of at least 6 individual aggregates analyzed in three independent experiments

Next, we studied the effect of the overexpression of *PCDHB15* on the aggregation of melanoma cells by monitoring the spontaneous formation of spheroids in the metastatic WM266-4 cell line and the three clones. The size and kinetics of the formation of the spheroids were studied by bright-field video microscopy. As early as 2 h after seeding, WM266-4 cells gathered and formed round aggregates with cell-to-cell interaction that strengthened with time (Fig. 3A). In contrast, cells overexpressing *PCDHB15* formed loose aggregates with different kinetics and maintained irregular shapes over time, suggesting a reluctance to engage straight contacts (Fig. 3A, B).

### PCDHB15 overexpression impairs melanoma cells 3D invasion

**Fig. 4:**
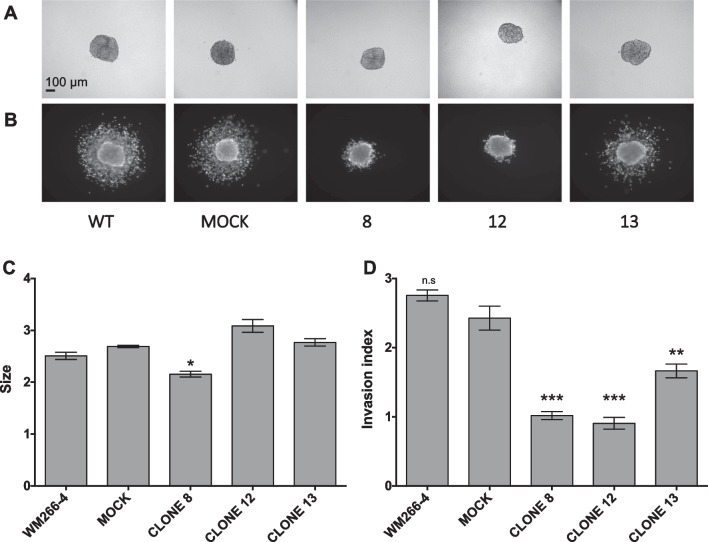
PCDHB15 overexpression in WM266-4 cells impairs 3D cell invasion. The invasion ability of WM266-4 cells (WT), control cells (mock) and PCDHB15-overexpressing cells (clones 8, 12 and 13) was measured using a 3D invasion assay in collagen matrix. Images are representative of at least 6 spheroids per condition before (**A**) and after (**B**) 24 h invasion. The initial sizes of each spheroid (**C**) and their invasion index at 24 h (**D**) are reported as histograms. Means and SEM were calculated from 6 spheroids measured in three independent experiments. Jarque–Bera’s test to analyze normality, Fisher’s test to analyze variances and Student *t* test were performed; *p* value < 0.05, **: *p* value < 0.01. ***: *p* value < 0.001

Another feature of the metastatic WM266-4 cell line is its 3D invasion ability, as we demonstrated previously [20]. After 72 h of culture in non-adherent conditions, WM266-4 cells spontaneously formed spheroids that were included in a collagen matrix (Fig. 4A). Invasion in the collagen matrix was measured after 24 h (Fig. 4B). Noteworthy, WM115 cells formed highly cohesive spheroids, but had no invasion capacity under these conditions. After collagen inclusion, the overexpression of *PCDHB15* had little effect on the spheroid size (Fig. 4C), but significantly reduced the invasive properties of WM266-4 cells (Fig. 4D). Interestingly, the greatest effect was observed with the two cell lines (#8 and #12) producing intermediate protein levels.

### PCDHB15 overexpression impairs lung metastasis formation in mice

**Fig. 5:**
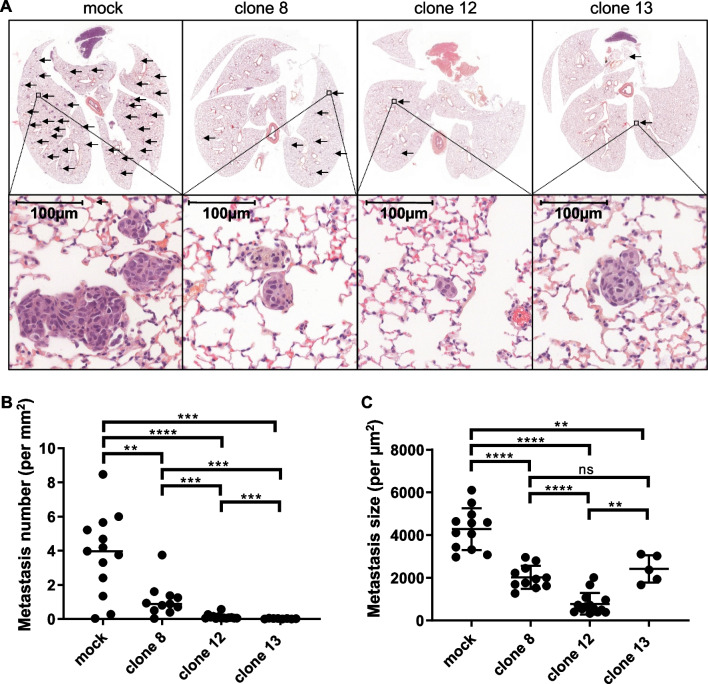
PCDHB15 overexpression impairs WM266-4 lung metastasis formation in vivo. WM266-4 overexpressing PCDHB15 (clone 8, 12, 13) or mock cells (with void vector) were injected in the tail vein (IV) of SCID mice. Lungs were recovered for immunohistochemical analysis 21 days after injection. **A** A representative image of the stained lung is shown for each group. Black arrows indicate metastases. Plots representing the median of number (**B**) and size (**C**) of metastases on one slice for each mouse are shown**.** *n* = 15 for mock, clone 8, clone 12; *n* = 14 for clone 13. Medians and SEM are shown. Mann–Whitney test; **p value < 0.01, ***p value < 0.001

The inhibitory effect of *PCDHB15* overexpression on in vitro melanoma cell aggregation and invasion led us to investigate the capacity of *PCDHB15* expressing melanoma cells to form lung metastasis in mice after intravenous injection as does the metastatic WM266-4 cell line [20]. We compared the effect of the three WM266-4 clones overexpressing *PCDHB15* to cells stably transfected with a void construct (mock cells). Immunohistochemical analysis of lungs 21 days after injection showed a dramatic decrease in lung metastasis formation with cells overexpressing *PCDHB15* compared to mock control WM266-4 cells (Fig. 5A). This result was confirmed by statistical analysis of the number (Fig. 5B) and size (Fig. 5C) of metastases, showing that the overexpression of *PCDHB15* reduces in vivo the invasion capacities of metastatic melanoma cells.

## Discussion

Melanoma generally evolves in a stepwise manner from initial benign or dysplastic *nevi* to metastatic melanoma, via two intermediate phases, the radial (RGP) and the vertical growth (VGP) phases [38, 39]. To characterize the extent and nature of DNA methylation modifications through melanoma progression, we have compared the DNA methylation profiles of melanoma cell lines representative of different aggressiveness status and focused our interest on genes that were hypermethylated in the most aggressive cell lines. This revealed the role of DNA methylation in the regulation of the mir199-A2 which down-regulation confers invasive traits in melanoma [20]. More recently, by comparing the genomic repartition of DNA methylation in cell lines of different aggressiveness status, we identified clusters of DNA hypermethylation that characterizes melanoma aggressiveness and, in particular, the gene *PCDHB15* [21]. *PCDHB15* belongs to the protocadherin *β* gene cluster located on chromosome 5q31. The clustered protocadherins *α*, *β*, and *γ* were mostly studied as putative neural receptors [40–42] that mediate the synaptic adhesive code between neurons in synaptogenesis. Stochastic single-neuron expression of clustered protocadherin protein isoforms by a mechanism involving alternative promoter choice [43] generated distinct cell surface identities [44, 45]. In the human central nervous system, the expression patterns of the PCDH-β genes are similar to those of the PCDH-α and PCDH-γ genes and contain 16 genes and 3 pseudogenes [42]. Each sequence corresponds to a single variable region exon encoding an extracellular domain with six characteristic cadherin ectodomain repeats (EC1-6), a transmembrane domain and an intracellular domain. All three types of protocadherins-*α*, -*β*, -*γ* can engage in isoform-specific trans-homophilic interactions [46]. They mediate neural self-recognition and non-self-discrimination. Interestingly, although classified as adhesion molecules, protocadherin homophilic interactions trigger neurite self-avoidance [47] that prevents interactions of axons and dendrites from the same neuron during development. However, the functional role of *PCDH* genes in tissues other than the brain is poorly explored. Several reports in the literature pointed toward a potential role of protocadherins as tumor suppressors in several cancers [48]. Considering that neurons and melanocytes are derived from the same embryonic tissue, these findings prompted us to characterize the functional role of PCDHB15 in cutaneous melanoma cells.

We showed that *PCDHB15* is strongly DNA hypermethylated at the 5′ end of its single exon, in the most aggressive melanoma cell lines compared to the less aggressive ones, as well as in the metastases compared to the corresponding primary melanomas. In cell lines, DNA hypermethylation of *PCDHB15* was associated with lower expression, which was reversed upon treatment with the demethylating drug 5azadC. Of note, the demethylation by 5azadC reached a plateau at 55%, probably meaning that all the accessible cytosines in the DNA sequence were replaced by 5azadC. Interestingly, a negative correlation between PCDHB15 promoter methylation and PCDHB15 expression was also reported in breast cancer [49]. Nevertheless, whereas these data strongly pointed out the role of DNA methylation in the regulation of *PCDHB15* expression, the direct involvement of the methylation in the regulatory regions at the 5′ end of the gene remains to be confirmed. To study the correlation, several approaches can be used as a CpG-free luciferase reporter vector system [50] or CRISPR/Cas9-mediated epigenetic edition [51]. Here, we evaluated whether the treatment with the demethylation agent, 5azadC, at low doses reverts *PCDHB15* silencing, as we have shown that 5azadC treatment at low doses reverted melanoma cell invasion in 3D invasion assays and in vivo metastasis formation [20]. We observed that *PCDHB15* expression was up-regulated by 5azadC treatment, in support of a regulatory role of DNA methylation at its promoter.

The stable overexpression of *PCDHB15* in cells, in which *PCDHB15* is silenced by DNA hypermethylation, dramatically impaired their aggregation capacity suggesting a non-adhesive role for PCDHB15 in agreement with a self-avoidance process as described for neurons [47]. Protocadherins *β* harbor extracellular cadherin motifs able to interact homophilically in trans, but how their truncated intracytoplasmic domain translates into the alteration of cellular adhesion remains to be understood [22]. Lower aggregation upon *PCDHB15* overexpression is associated with impaired 3D invasiveness, suggesting the potential importance of an aggregative behavior in the invasive abilities of melanoma cells. This is in agreement with the reported lower cancer cell dissemination when tumor cells migrate as individual cells compared to aggregated cells [52]. Taken together, these in vitro effects suggest that silencing of *PCDHB15* in melanoma cells participates in the fine-tuning of the aggregative behavior of melanoma cells during melanoma progression and favors specific metastatic properties. The in vivo experiment confirmed the in vitro findings, showing that the overexpression of *PCDHB15* impairs the formation of lung metastases in mice. Of note, whereas the three *PCDHB15*-expressing cell lines showed similar tendencies in the different functional assays, they did not highlight a strict correlation between the levels of PCDHB15 expression and their inhibitory effects, compared to the parental cell line. One cannot exclude that high expression levels of the protein could alter its proper processing as well as its cellular function. Nevertheless, the obtained data parallel what has been observed in breast cancer cell lines, in which overexpression of other members of the PCDH-β gene family (*PCDHB4* and *PCDHB19)* inhibited anchorage-independent cell growth in soft agar, colony formation ability and in vivo tumor growth in NOD/SCID mice [53].

In concordance with our findings, *PCDHB15* was identified as a part of a specific methylation signature across breast and colon cancer [54], as *PCDHB13* in Non-Small Cell Lung Cancer [55]. A functional role for the hypermethylation and gene silencing of PCDHαβγ family genes (*PCDHAC2*, *PCDHB7*, *PCDHB15*, *PCDHGA1* and *PCDHGA6*) was also identified recently in colorectal cancer influencing the WNT/B-catenin pathway implicated in proliferation, survival and migration [56]. More recently, *PCDHB15* was proposed as a potential tumor suppressor in breast cancer, based on the observation of a positive correlation between PCDHB15 expression and relapse-free survival [49]. Interestingly, ectopic expression of *PCDHB15*, which is down-regulated by DNA methylation in the MDA-MB-231 breast cell line, suppressed colony formation.

## Conclusions

In this study, we demonstrate an epigenetic regulation of the expression of the *PCDHB15* gene in melanoma cell lines. This gene is silenced in metastatic cells, and its stable overexpression reduced cell aggregation and invasion capacity in vitro and in vivo. Taken together, our data suggest for the first time a potential role of tumor suppressor for *PCDHB15* in melanoma. Mechanisms by which PCDHB15 may play a role in aggregation and invasion are to be further studied. In accordance with findings in other cancers, we propose that the role of the protocadherin genes and their interactions in cancer progression will be an area of interest to investigate in the future.

## Data Availability

Not applicable.

## Abbreviations

5azadC: 5Aza-2′-deoxycytidine
AJCC: American Joint Committee on Cancer
CIMP: CpG island methylator phenotype
CpG: Cytosine preceding guanine nucleotide dimer 5′-3′ direction
DNA: Deoxyribonucleic acid
DOM: Distant organ metastasis
LNM: Lymph node metastasis
ncRNA: Noncoding ribonucleic acid
PBS: Phosphate-buffered saline
PCDHB: Protocadherin beta
PRM: Primary tumor
